# Shared emotions, interpersonal syntonization, and group decision-making: a multi-agent perspective

**DOI:** 10.3389/fnins.2023.1251855

**Published:** 2023-11-30

**Authors:** Davide Crivelli, Michela Balconi

**Affiliations:** ^1^International research center for Cognitive Applied Neuroscience (IrcCAN), Faculty of Psychology, Università Cattolica del Sacro Cuore, Milan, Italy; ^2^Research Unit in Affective and Social Neuroscience, Department of Psychology, Università Cattolica del Sacro Cuore, Milan, Italy

**Keywords:** group decision-making, emotion, interpersonal syntonization, shared representations, inter-agency, hyperscanning

## 1 Emotions across individual and group decision-making

Group decision-making refers to the process of making decisions collectively, involving multiple individuals who work together to discuss, analyze, and choose among various alternatives. It plays a crucial role in many domains, including business, politics, organizations, and social interactions. Research has demonstrated that group decision-making can yield outcomes that are superior in quality and creativity compared to individual decision-making (Paulus and Nijstad, 2003). This is because groups offer diverse viewpoints, increased information pooling, and the potential for synergistic interactions. Here, we would like to point out how the complex process of group decision-making involves—in addition to common background knowledge, intentions, and joint actions—the coordination of multiple individuals' emotions and shared representations (Balconi and Fronda, 2020a), respectively understood as the complex integration of physiological reactions, subjective experience, and affective drives, and as jointly created and shared internal images (though not necessarily visual and often multimodal) of aspects of the external reality or related interpretations and abstractions (e.g., external objects/agents but also, as an example, mental states and behaviors).

The notion that situated emotional responses can have an impact on, influence, and even forecast wise and advantageous decisions is—to date—considered a fact, with these responses representing a powerful tool for decision-making (Morelli et al., 2022). Embodied cognition models provide the background for such remarks, with the idea that emotions and, especially, physiological responses related to affective experience play a role as informational input for decision-making and other cognitive functions (Pace-Schott et al., 2019).

Narrowing the focus to the positive and pivotal role of emotions in decision-making under risk, Slovic et al. (2004, 2005) postulated the so-called “affect heuristic”. Under this view, affects inform the process of distinguishing a stimulus's positive or negative connotation and the attribution of “goodness” or “badness” perceived as a feeling. Notably, such perceptions may occur both with and without consciousness. According to the authors, the feelings that emerge during a judgment or decision-making process rely on the characteristics of the person and the activity as well as on how these two factors interact, evoking emotion-laden representations. Since it is easier and more effective to use an overall, readily available affective impression in complex or resource-constrained situations, affect is here considered as a shortcut or a heuristic (Finucane et al., 2000). Nevertheless, the affect heuristic fails to recognize that decisions driven by affect can diverge from cognitive evaluation and how such divergence can affect behavior.

Trying to overcome such limitations, the risk-as-feeling hypothesis of decision-making under risk (Loewenstein et al., 2001) postulates that behavior and responses to risky situations and decision-making are determined by the interplay between emotional reactions to risk and its cognitive evaluation. Furthermore, the hypothesis moves from the assumption that both anticipated feelings concerning potential consequences of risky choices and anticipatory feelings actually connoting the affective experience during the decisional process (e.g., fear, anxiety, worry, and excitement) specifically connote such a process and determine its outcome. The modulatory role of anticipated and anticipatory feelings in decision-making under risk is grounded in the physiological correlates of such feelings, which enter information processing as afferent embodied signals. Emotions and related physiological responses, then, would not contribute to decisions only by delineating shortcuts, but also contribute to information processing, and both influence and are influenced by cognitive appraisal, individual interpretation of the situation, and situational factors.

Notably, those very same emotional responses—in both their explicit behavioral and implicit physiological manifestations—can be influenced by various factors, including—beyond the framing of information and personal experiences—micro/macro cultural background and social group influences. Moreover, group decision-making involves considerations of fairness, trust, and cooperation, which are also inherently linked to emotions. Affects such as empathy, trust, guilt, and moral outrage can, indeed, influence how individuals evaluate risks and make decisions in social contexts as either upregulating or downregulating factors. For instance, some people might be more likely to take risks or engage in altruistic behavior when motivated by feelings of compassion or empathy toward others, even beyond individual benefit (Balconi and Canavesio, 2013; Balconi and Fronda, 2020b; Balconi et al., 2020).

Social contexts and group settings may thus exacerbate or amplify the influence of emotions on decisional processes, especially when situational factors such as perception of risk or threat, informational overload or ambiguity, and time pressure increase the psychological and cognitive load on decisions. Neuroimaging studies have revealed that emotional processing during group decision-making engages brain regions involved in affective responses, such as the amygdala and the insula (Phelps et al., 2014; Balconi, 2020; Van Kleef and Côté, 2022). The activation of the amygdala reflects the role of emotional salience, while the role of the insula is primarily linked to the processing and creation of active inferences concerning interoception and empathic responses (Barrett and Simmons, 2015; Crivelli and Balconi, 2023). Furthermore, feedback received from co-agents plays a crucial role in shaping group decision-making, and neuroscientific investigations have shed light on its neural correlates. Studies investigating neurofunctional activity have shown that receiving social feedback, such as agreement or disagreement from other group members, elicits distinct neural responses (Balconi and Vanutelli, 2017). The processing of social feedback involves brain regions associated with reward and social evaluation, such as the ventral striatum and the anterior cingulate cortex (Kishida and Montague, 2012; Sobczak et al., 2023). These findings suggest that the brain integrates social information during group decision-making, influencing subsequent decision processes and collective actions.

While the summative role of individual affective experience and psychophysiological responses for single co-agents constituting a decisional group dealing with a risky decision might still be accounted for and investigated in light of embodied cognition models such as the risk-as-feelings hypothesis (Loewenstein et al., 2001) and the neurovisceral integration model (Thayer and Lane, 2000), we posit that at least one more factor should be taken into account to better understand (and perhaps to assess and improve) this complex phenomenon: the emergence of shared representations, affective experience, and intentions shaping group relational dynamics.

## 2 Making the picture bigger: a multi-agent perspective on shared affects and interpersonal syntonization in group decision-making

Moving from a definition of joint actions as “social interactions wherein two or more individuals coordinate their actions in space and time to bring about a change in the environment” (Knoblich and Sebanz, 2006, p. 100), we here propose that group decision-making can be considered as a special case of such a form of social interaction, which—while still implying the sharing of action representations and the integrative coordination of co-agents' actions to achieve common goals (Pacherie and Dokic, 2006; Sebanz et al., 2006; Crivelli and Balconi, 2010)—shapes its purpose of bringing about a change in the environment in the form of a dynamic sense-making process aimed at defining a shared interpretation of the situation, at shaping a shared action plan, and at selecting what seem to be the best decisional option in such situation. Thus, ensuring the successful implementation of the decision requires planning, coordination, and the allocation of responsibilities. Group members need to commit to the decision, understand their roles, and work collectively toward its execution.

Within this framework, shared mental representations of situations, goals, opportunities for action, and commitments within the group shape the direction and coherence of group decision-making processes (Gilbert, 1990) and provide a common framework for understanding and coordinating actions, ensuring that group members align their efforts toward a common goal, thus developing *collective intentions* and a sense of *inter-agency* (Crivelli and Balconi, 2010, 2017b). Notably, inter-agency and collective intentions emerge from the exchange and integration of individual intentions, beliefs, and interpretations of the situation—including their perceptions of the affective connotation of such a situation—leading to a shared understanding and commitment (Tuomela, 2013). In our view, such integration and sharing set the stage for the emergence of a collective decisional unit—i.e., a multi-agent decisional entity—which, if effective, would be able to deliver properly collective decisional processes and reach group decisional outcomes. Group decision-making can actually express itself in all its complexity only if delivered by a unified decisional unit. The definition of multi-agent decisional entity we propose is, then, not merely the sum of individual decision-makers sharing a task but a unified collective entity constituted by multiple agents involved in a decisional task or situation that, in addition, acts based on shared background knowledge and co-created representations, on collective intentions and joint-action plans, and on shared agency. From this perspective, social interactions are not merely something accompanying decisional processes but are the vessels for such a collaborative sense-making process, which occurs via both explicit discussions and implicit cues, such as, again, affective non-verbal communication. The intrinsic social–affective connotation of group decision-making and of the shared sense-making process introduced above is further highlighted by the recognized contribution of social skills—such as perspective-taking, mentalizing, and social reasoning—in facilitating the integration of individual perspectives and the development of shared mental representations (Tomasello et al., 2005) as well as by the contribution of socio–motivational processes—including social identification, trust, and cooperation—to the commitment and adherence to collective intentions (Frith and Singer, 2008; Haslam et al., 2020).

**Figure 1:**
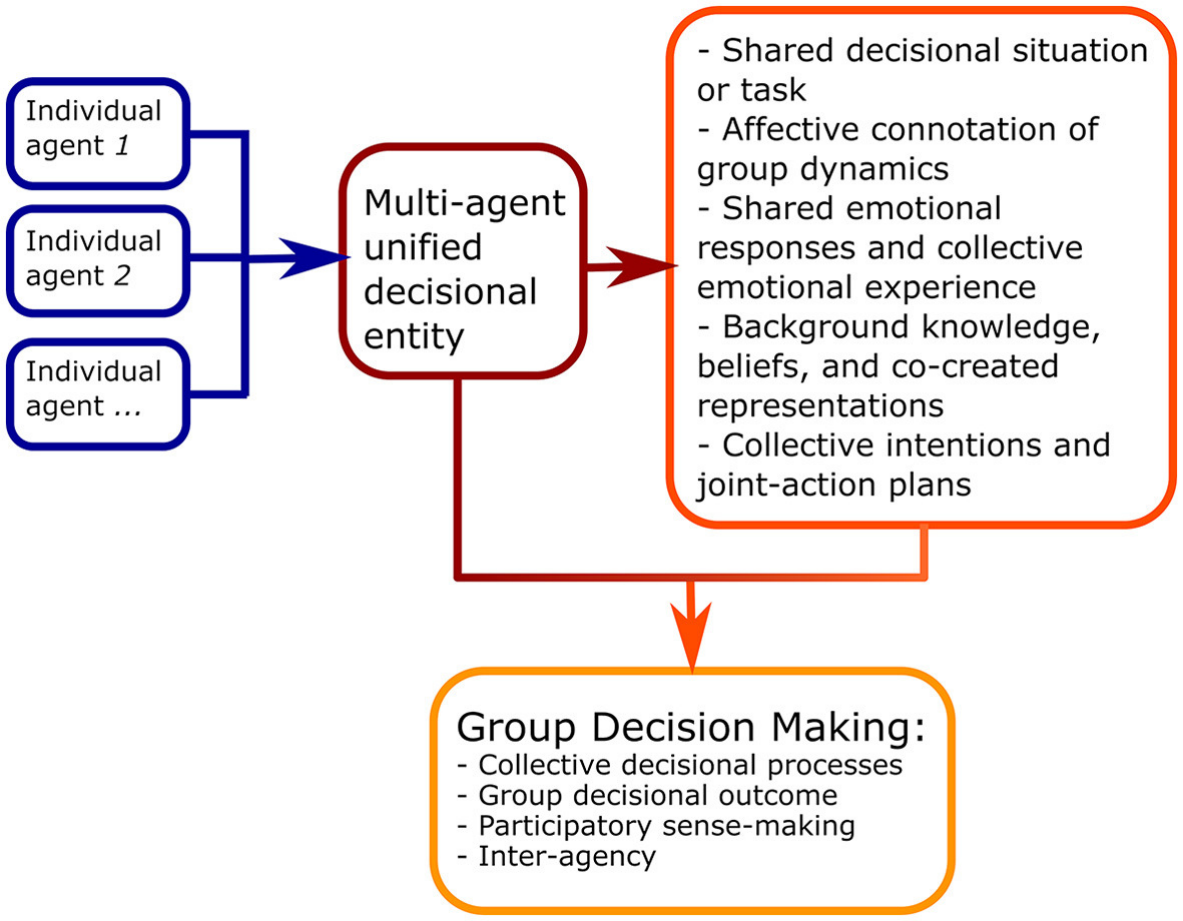
Schematic representation of the proposed multi-agent perspective on shared affects and interpersonal syntonization in group decision-making.

Drawing a parallel with traditional embodied models of decision-making and, in particular, with the risk-as-feeling hypothesis (Loewenstein et al., 2001), we here build on those remarks and propose that, when decisional processes include multiple agents working together as a genuinely unified decisional entity, the affective connotations of the group dynamics and collective emotional experience deriving from the decisional process itself affect the outcomes of such a process, as well as the quality of information-processing steps leading to those outcomes. These emerging collective components enter the decisional process and, thus, monitoring them becomes strategic in properly understanding how successful vs. unsuccessful group decision-making evolves (Figure 1). Being related to psychological and relational processes leading to the creation of shared experiences—including sharing of emotional responses (i.e., empathic resonance), sharing of intentions and action plans (i.e., mirroring), and sharing of beliefs and representations (i.e., a common vision)—interpersonal syntonization and synchronization of psychophysiological activity among group members might act as a valuable and quantifiable marker of such processes emerging from social interaction. Such a marker could provide a complementary and more finely graded view; it may integrate traditional approaches to study collective decisional processes delivered by multiple interacting agents and, more generally, joint tasks, such as the investigation of collective efficacy in shared tasks via self-report tools, analysis of occurrence of synchronized behavioral patterns and implicit mimicry among co-agents, or the emergence of co-constructed semantic units in verbal communication.

Functional magnetic resonance imaging (fMRI) studies have demonstrated that when individuals engage in joint decision-making, there is an increase in neural synchronization in brain regions involved in social cognition, such as the prefrontal cortex and the mirror neuron system (Frith and Singer, 2008; Hou et al., 2022; Zhao et al., 2023). This synchronization has consistently been interpreted as a marker of processes that facilitate mutual understanding and shared mental representations among group members, promoting effective communication and collaboration. Similarly, it has been shown that prefrontal structures supporting attention regulation, social responsiveness, and self-regulation show greater activation when a social frame is added to a motor synchronization task and when co-agents are asked to focus on the modulation of interoceptive feelings that occur during the joint task (Angioletti and Balconi, 2022; Balconi and Angioletti, 2023).

With the introduction of the hyperscanning approach (Montague et al., 2002; Babiloni and Astolfi, 2014; Balconi and Molteni, 2016; Balconi et al., 2017; Crivelli and Balconi, 2017a), a novel perspective was introduced in social neuroscience. Hyperscanning involves the use of psychophysiological techniques, such as functional magnetic resonance imaging (fMRI), electroencephalography (EEG), functional near-infrared spectroscopy (fNIRS), and/or autonomic recording (e.g., electrodermal and cardiovascular activity), to record physiological activity from multiple individuals involved in a shared task and the subsequent integrated analysis of the resulting data. In the last 2 decades, this novel approach has revolutionized the way group dynamics can be studied and has been progressively refined and made more and more usable even in real-life settings, and it is now ready to offer concrete solutions for qualification and quantification of interpersonal syntonization and emotional tuning across co-agents involved in realistic social exchanges (Balconi and Molteni, 2016; Balconi and Fronda, 2020a; Balconi et al., 2022). Even if the literature on group/shared decisional processes is currently still in progress, a few consistent pieces of evidence have already been outlined. Specifically, since the first attempts to investigate neurophysiological markers of interpersonal syntonization during shared complex tasks involving decision-making, the role of cortical structures in supporting mentalization and general social understanding has been observed. As an example, Zhang et al. (2017), via an fNIRS hyperscanning paradigm based on a face-to-face gambling game, observed increased inter-brain coherence in the medial and dorsolateral prefrontal cortices of playing dyads. Similarly, Hu et al. (2018) reported larger theta and alpha inter-brain synchrony, respectively, over fronto-central and centro-parietal sites, in a high-cooperation condition during an interactive prisoner's dilemma task. Similarly, Jahng et al. (2017) discussed the role of the right temporoparietal region and the presence of inter-brain synchrony in the alpha range as a marker of strategic planning in deciding whether to cooperate or defect in an iterated version of the prisoner's dilemma. More recently, research has begun to focus on proper shared decision-making tasks. Zhang et al. (2021), for example, used a multi-person version of the prisoner's dilemma to investigate team collaborative decisional processes. They observed higher inter-brain synchrony in the right inferior frontal gyrus during a collaborative decision-making condition connoted by high-incentive rewards. Additionally, gradual group polarization processes (i.e., risky shift and cautious shift in risky group decision-making) have been found to be associated with enhanced inter-brain synchronization of bilateral prefrontal areas and the left temporoparietal junction between inter-agents (Hou et al., 2022). Interestingly, Zhao et al. (2023) have recently suggested that group decision-making under uncertainty (investigated via a turn-based Balloon Analog Risk Task) may be affected by the interpersonal relationship (i.e., interacting with a friend vs. stranger) between co-agents and social value orientation. Specifically, fNIRS-based inter-brain synchronization in the left inferior frontal gyrus and medial frontopolar cortex was modulated by interpersonal relationships.

## 3 Conclusion

Understanding the interrelation between relational dynamics, the emergence of shared affective experiences, the formation of shared mental representations, and group decision-making has, on the one hand, practical implications in various domains. In organizational settings, it can indeed inform the design of collaborative processes, team-building strategies, and leadership development programs (Choi and Kim, 1999; Uitdewilligen and Waller, 2018; Zhu et al., 2021). In social movements, it can shed light on how collective intentions mobilize collective action and promote social change (van Zomeren et al., 2008). On the other hand, such understanding may help in preventing the dark side of group thinking. Indeed, research suggests that when groups develop a shared identity or group norm that supports risk-taking, shared affects and representations may align with engaging in risky behavior (Van Vugt et al., 2004; Harth et al., 2013). Furthermore, group discussions and interactions may amplify individuals' initial inclinations toward risk-taking behavior, a phenomenon classically known as risky shift (Clark, 1971).

While it is still evolving, at least in its practical implications, we here propose that the hyperscanning approach (Montague et al., 2002; Babiloni and Astolfi, 2014; Balconi and Molteni, 2016; Crivelli and Balconi, 2017a) may already be used as a methodological frame for formal investigation of implicit processes leading to the creation of collective affective experience; of their particular role in shaping neurofunctional markers of interpersonal syntonization among co-agents in decisional groups; and—ultimately—of the influence of such factors on group decisional processes.

## Author contributions

DC wrote the first draft of the manuscript and MB critically revised it. Both authors contributed to the conception of the present work, read, and approved the submitted version.
